# *hOGG1*基因Ser326Cys多态性与肺癌易感性的*meta*分析研究

**DOI:** 10.3779/j.issn.1009-3419.2011.03.05

**Published:** 2011-03-20

**Authors:** 乾 钱, 仍允 刘, 哲 雷, 嘉琮 尤, 清华 周, 洪涛 张

**Affiliations:** 1 215123 苏州，苏州大学癌症分子遗传学实验室 Soochow University Laboratory of Cancer Molecular Genetics, Medical College of Soochow University, Suzhou 215123, China; 2 300052 天津，天津医科大学总医院，天津市肺癌研究所，天津市肺癌转移与肿瘤微环境重点实验室 Tianjin Key Laboratory of Lung Cancer Metastasis and Tumor Microenvironment, Tianjin Lung Cancer Institute, Tianjin Medical University General Hospital, Tianjin 300052, China

**Keywords:** *hOGG1*, 肺肿瘤, 易感性, *Meta*分析, *hOGG1*, Lung neoplasms, Susceptibility, *Meta*-analysis

## Abstract

**背景与目的:**

就全世界范围内而言，肺癌是一种常见疾病。人8-羟基鸟嘌呤糖苷酶（human 8-hydroxyguanine glycosylase, hOGG1）是一种DNA修复酶，它能特异切除8-羟基鸟嘌呤（8-dihydro-8-oxoguanine, 8-OH-G），对损伤的DNA进行修复。*hOGG1* Ser326Cys基因多态性与癌症易感性的关系一直是研究的热点，而该多态性与肺癌易感性的关系尚存在争议。本研究采用*meta*分析旨在更好地探讨*hOGG1* Ser326Cys多态性与肺癌易感性之间的关系。

**方法:**

使用MEDLINE数据库检索2010年11月以前的相关文献，按照纳入标准，全面搜索含有研究*hOGG1* Ser326Cys多态性与肺癌易感性相关的信息。由至少两位评论员做独立文献筛选和资料提取，并交叉审核。使用STATA 10.1软件进行统计分析。

**结果:**

根据检索条件，共有22篇文献（包括8, 575例肺癌患者和9, 484名正常对照个体）被纳入当前的*meta*分析。分析结果表明22项研究的结果存在明显异质性，当排除不符合*Hard-Weinberg*平衡定律的两篇文献后，其余的文献呈现出较好的同质性。与*hOGG1* Ser326相比，Cys326基因型明显增加了肺癌发病风险（OR=1.24, 95%CI:1.10-1.39, *P* < 0.001）。这种正相关在亚洲人群和医院来源的样本中尤为明显（OR=1.28, 95%CI: 1.11-1.49; OR=1.26, 95%CI: 1.09-1.46）。

**结论:**

*hOGG1* Ser326Cys多态性与肺癌易感性之间存在明显相关性，Cys326基因型能明显增加肺癌发病风险。

## Abstract (en)

**Background and objective:**

Human 8-hydroxyguanine glycosylase (*hOGG1*) is one of the DNA repair genes, which can repair damaged DNA by specifically excising 8-dihydro-8-oxoguanine (8-OH-G). A considerable number of studies investigating *hOGG1* Ser326Cys polymorphism were in relation to various cancers. However, the association of *hOGG1* Ser326Cys polymorphism with risk of lung cancer is inconsistency. The aim of this study is to assess the association of *hOGG1* Ser326Cys polymorphism with risk of lung cancer by conducting a *meta*-analysis.

**Methods:**

To identify all studies that are qualified for *meta*-analysis, we conducted a computerized literature search of MEDLINE database (before November, 2010). Two investigators independently extracted the data and reached a consensus on all items.

**Results:**

According to our search limits, twenty-two case-control studies, including 8, 575 lung cancer cases and 9, 484 controls, were selected for *meta*-analysis. Significant heterogeneity was found among those twenty-two case-control studies, when excluding two studies that were not accorded with *Hard*-*Weinberg* Equilibrium, the remains performed well homogeneity. Compared with Ser326 genotype, Cys326 genotype can significantly increase risk of lung cancer (OR=1.24, 95%CI: 1.10-1.39, *P* < 0.001), especially in Asian ethnic population and hospital population (OR=1.28, 95%CI: 1.11-1.49; OR=1.26, 95%CI: 1.09-1.46).

**Conclusion:**

The present study indicates that the *hOGG1* Ser326Cys polymorphism is associated with risk of lung cancer, Cys326 genotype can significantly increase risk of lung cancer.

癌症的发生是一个复杂的逐步积累的过程，在这个过程中正常细胞遗传信息发生改变从而导致表型改变，进一步使其具有入侵和转移到其它部位的能力，这是导致癌症病人死亡的主要原因之一^[1]^。另外，癌症的发生与环境因素也有着直接的联系，吸烟便是一类众所周知的致癌因素。严重的致癌环境能导致多种DNA损伤，如DNA的氧化损伤^[2]^。人8-羟基鸟嘌呤糖苷酶（human 8-hydroxyguanine glycosylase, hOGG1）是一种DNA修复酶，它能特异切除8-羟基鸟嘌呤（8-dihydro-8-oxoguanine, 8-OH-G），对损伤的DNA进行修复^[3]^。从遗传的角度来说，倘若DNA修复基因的遗传变异能影响其对DNA的修复能力，这便意味着癌症发病风险的增加。

大量的分子流行病学研究对*hOGG1*基因多态性与各种癌症易感性进行了分析，这些研究大都集中在*hOGG1*基因的功能多态性位点与肺癌、肝癌和乳腺癌的相关性方面，尤以Ser326Cys最为常见^[4]^。该位点存在于*hOGG1*基因第7外显子的第1245位碱基处（1245C/G），由于C/G多态使第326位密码子可编码丝氨酸（ser，密码子TCC）或编码半胱氨酸（cys，密码子TGC），形成hOGG1-Ser326和hOGG1-Cys326两种蛋白，后者导致糖苷酶活性降低，致使hOGG1修复8-OH-G的能力低下，造成8-OH-G在细胞内的残留，从而增加了基因突变和癌症的风险。然而关于Ser326Cys多态性与肺癌易感性关系的研究结果并不一致^[3, 4]^，为了更好地探讨*hOGG1*多态性与肺癌易感性之间的关系，我们收集、整理既往研究数据，并进行*meta*分析。

## 1 材料与分析

### 1.1 数据收集

将“human 8-hydroxyguanine glycosylase”、“hOGG1”、“polymorphisms”、“genetic variation”、“lung cancer”、“lung carcinoma”等作为关键词，使用MEDLINE数据库检索2010年11月以前的相关文献。在检索到的文章全文中仔细搜索是否含有hOGG1 Ser326Cys多态性与肺癌易感性相关的信息。

### 1.2 纳入标准

包括：①运用相互独立的病例-对照试验设计；②基因多态性对应的基因型频率可以从文献中获取。

两位研究者（钱乾和刘仍允）通过独立阅读所获得文献题目和摘要，在排除明显不符合纳入标准的文献后，对可能符合纳入的文献阅读全文，以确定是否真正符合纳入标准，并交叉核对纳入文献的结果，且对有分歧的文献通过讨论或由第3个研究者（雷哲）决定其是否纳入。

### 1.3 数据整理及统计分析

对于符合纳入标准的每一篇文献均按照作者、发表年份、试验人群的来源和人种，以及患者和对照组中各基因型携带者的数量进行整理（表 1）。采用*Person*拟合度卡方检验分析对照组基因型分布是否符合*Hard-Weinberg*平衡（HWE）。使用以*Chi*^2^检验为基础的*Q*检验衡量样本的同质性^[5]^，并以*I*^2^的大小反应多组研究之间同质性的程度（*I*^2^值越小，同质性越强）^[6]^。当样本间不具有明显异质性的时候，采用基于Mantel-Haenszel方法的固定效应模型分析^[7]^；反之，采用DerSimonian and Laird方法的随机效应模型分析。漏斗图（funnel plot）用来检测数据之间是否存在发表偏倚，其程度采用*Egger*检验进行计算。基因多态性与肺癌易感性的关系的强弱程度用OR值表示^[8]^，而OR值是否明显采用*Z*检验。上述统计分析均采用STATA软件（Stata/SE version 10.1 for Windows; Stata Corp, College Station, Texas），*P* < 0.05为有统计学差异。

**1:** 纳入*meta*分析的22项独立研究的基本特征 Characteristics of twenty-two studies included in the *meta* -analysis

Author	Year	Source of controls	Ethnicity	Cases				Controls			*P* for *HWE*
				CC	CG	GG		CC	CG	GG	
Khono^[12]^	1998	Population	Asian	16	19	10		15	20	7	0.939
Sugimura^[13]^	1999	Hospital	Mixed	85	115	41		63	107	27	0.082
Wikman^[14]^	2000	Hospital	Caucasian	68	32	5		60	43	2	0.067
Ito^[15]^	2002	Hospital	Asian	40	71	27		68	118	54	0.837
Le^[16]^	2002	Population	Mixed	123	110	65		177	175	53	0.350
Sunaga^[17]^	2002	Hospital	Asian	54	106	38		50	66	36	0.126
Lan^[18]^	2004	Population	Asian	37	61	20		51	43	15	0.232
Park^[3]^	2004	Hospital	Caucasian	101	65	13		255	87	8	0.857
Vogel^[4]^	2004	Population	Caucasian	149	93	14		159	91	19	0.237
Hung^[19]^	2005	Hospital	Caucasian	1, 401	661	93		1, 368	716	79	0.215
Liang^[20]^	2005	Hospital	Asian	154	69	4		158	66	3	0.178
Khono^[21]^	2006	Hospital	Asian	285	544	268		123	190	81	0.628
Loft^[22]^	2006	Population	Caucasian	144	93	14		154	88	19	0.200
Sorensen^[23]^	2006	Population	Caucasian	254	155	22		479	284	33	0.258
Zienolddiny^[10]^	2006	Population	Caucasian	182	100	44		194	117	75	< 0.001*
De Ruyck^[24]^	2007	Hospital	Caucasian	74	33	3		60	46	4	0.176
Hatt^[25]^	2007	Population	Caucasian	92	58	8		93	59	12	0.536
Karahalil^[26]^	2008	Population	Caucasian	86	65	14		115	106	29	0.546
Chang^[27]^	2009	Hospital	Asian	142	518	436		154	482	361	0.741
Miyaishi^[28]^	2009	Hospital	Asian	27	55	26		39	54	28	0.271
Okasaka^[29]^	2009	Population	Asian	117	257	141		250	544	236	0.070
Liu^[9]^	2010	Hospital	Asian	68	158	132		110	294	312	0.004^*^
^*^The studies significantly derived from *HWE* (*P* < 0.05).											

## 2 结果

根据检索条件，共有22篇文献^[3, 4, 9, 10, 12-29]^符合纳入标准（表 1）。共有8, 575例肺癌患者和9, 484名正常对照个体被纳入*meta*分析，其中10项研究（包括3, 900例患者和4, 028名对照个体）来源于亚洲人群，10项研究（包括4, 136例患者和4, 854名对照者）来源于高加索人群，另外2项研究（包括539例患者和602名对照者）的样本均源于两种以上的人群。

### 2.1 hOGG1 Ser326Cys多态与肺癌易感性

将收集到的22篇文献^[3, 4, 9, 10, 12-29]^全部纳入*meta*分析，发现hOGG1 Ser326Cys多态性与肺癌易感性之间并无明显相关性，然而这些文献之间存在很强的异质性（表 2）。按照人种或者对照来源进行分层分析，结果表明22篇文献间的异质性主要由高加索人或医院来源的研究所引起。与hOGG1 Ser326相比，Cys326基因型可以明显增加亚洲人群肺癌的发病风险（OR=1.17, 95%CI: 1.02-1.35, *P*=0.023），但是这种明显相关性在高加索人群中并不存在。

**2:** Ser326Cys基因多态性位点的基因型及其在肺癌中的OR值 Summary of genotyping for Ser326Cys polymorphism and their ORs in lung cancer

Variables	*n*	Cases/Controls	CG versus CC				GG versus CC				CG/GG versus CC		
			OR (95% CI)	*P*_H_^*^	*I*^2^(%)		OR (95% CI)	*P*_H_	*I*^2^(%)		OR (95% CI)	*P*_H_	*I*^2^(%)
Total	22	8, 575/9, 484	1.04 (0.94-1.14)	0.028	40.0		1.11 (0.94-1.31)	0.007	47.8		1.05 (0.94-1.16)	0.003	51.3
Ethnicities													
Asian	10	3, 900/4, 028	1.14 (1.01-1.28)	0.432	0.7		1.17 (1.02-1.35)	0.135	34.1		1.15 (1.03-1.29)	0.231	23.1
Caucasian	10	4, 136/4, 854	0.98 (0.84-1.15)	0.025	52.7		0.97 (0.71-1.34)	0.021	54.0		0.97 (0.82-1.14)	0.004	62.6
Mixed	2	539/602	0.86 (0.66-1.12)	0.641	0		1.51 (1.07-2.13)	0.224	32.3		1.01 (0.79-1.28)	0.334	0
Source of controls													
Pop.	10	2, 563/3, 712	1.02 (0.91-1.14)	0.593	0		1.04 (0.79-1.36)	0.023	53.2		1.02 (0.92-1.14)	0.315	13.9
Hosp.	12	6, 012/5, 772	1.05 (0.89-1.24)	0.004	60.1		1.16 (0.94-1.44)	0.038	46.6		1.06 (0.90-1.26)	0.001	66.3
^*^The presence of heterogeneity (*P*_H_ < 0.05).													

对纳入的22篇文献^[3, 4, 9, 10, 12-29]^的对照组基因型数据进行*HWE*检验，发现2篇文献^[9, 10]^的数据不符合*HWE*。其中一篇源于亚洲人群^[9]^，另一篇源于高加索人群^[10]^。将这2篇文献排除后发现其它20篇文献之间显示出较好的同质性（*P*=0.242, *I*^2^=17.0%），且与hOGG1 Ser326相比Cys326基因型明显增加了24%的肺癌发病风险（OR=1.24, 95%CI: 1.10-1.39, *P* < 0.001）（图 1）。按照人种进行亚组分析后，在亚洲和混合型人群中仍然可见这种与肺癌易感性的正相关现象（*P*=0.001, *P*=0.020）（图 1）。按照样本来源进行亚组分析的结果表明，在人群来源的研究和医院来源的研究中hOGG1 Cys326基因型较Ser326基因型均明显增加了肺癌易感性（*P*=0.05, *P*=0.002）（图 2）。

**1:**
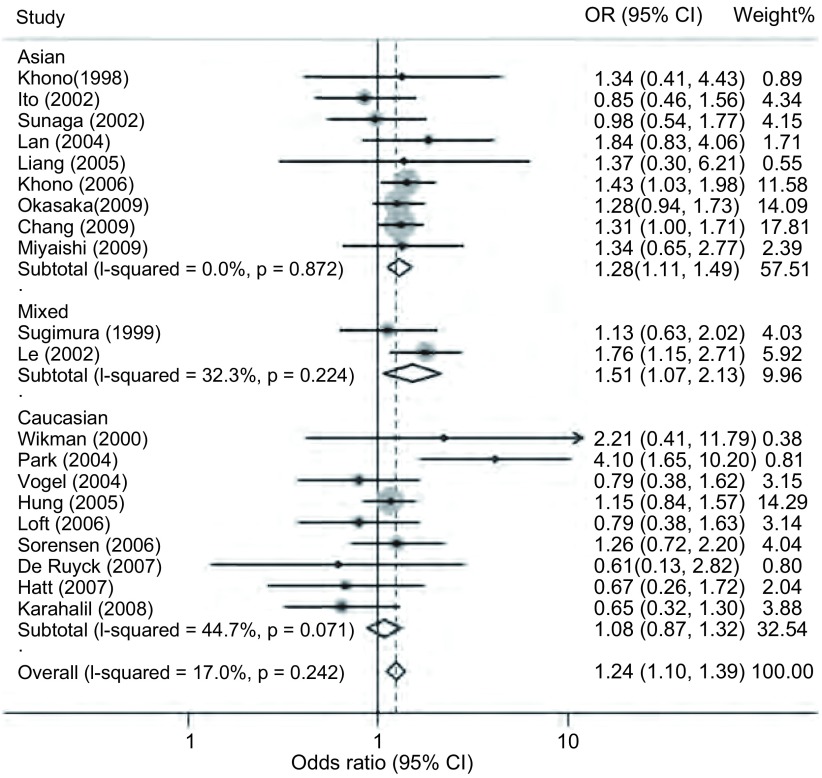
根据人种来源分组的Ser326Cys多态性位点在肺癌中的*meta*分析 *Meta* -analysis for Ser326Cys polymorphism in lung cancer by ethnic subgroup

**2:**
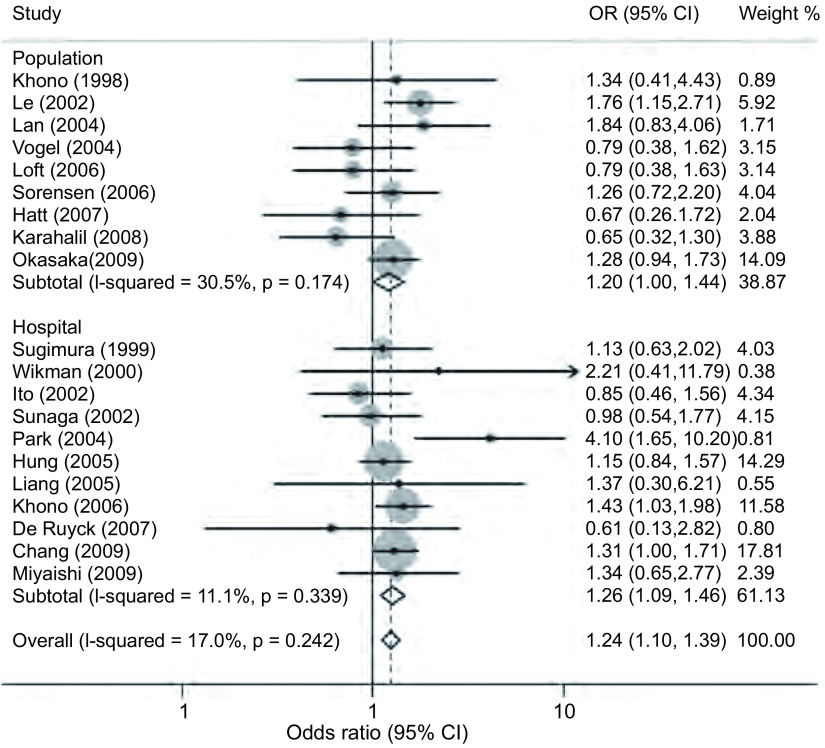
根据样本来源分组的Ser326Cys多态性位点在肺癌中的*meta*分析 *Meta* -analysis for Ser326Cys polymorphism in lung cancer by cohort subgroup

### 2.2 发表偏倚

使用OR值的自然对数及其标准误创建漏斗图检测发表偏倚，漏斗图表现出良好的对称性，说明本*meta*分析并不存在发表偏倚（图 3）。此外，*Egger*检验结果证实了漏斗图的结果（*t*=-0.23, *P*=0.823）。亚组分析后发现在亚洲人群和高加索人群中均不存在发表偏倚（*t*=-0.06, *P*=0.955; *t*=0.20, *P*=0.847），人群来源的研究和医院来源的研究也不存在明显的发表偏倚（*t*=-0.70, *P*=0.506; *t*=0.34, *P*=0.739）。

**3:**
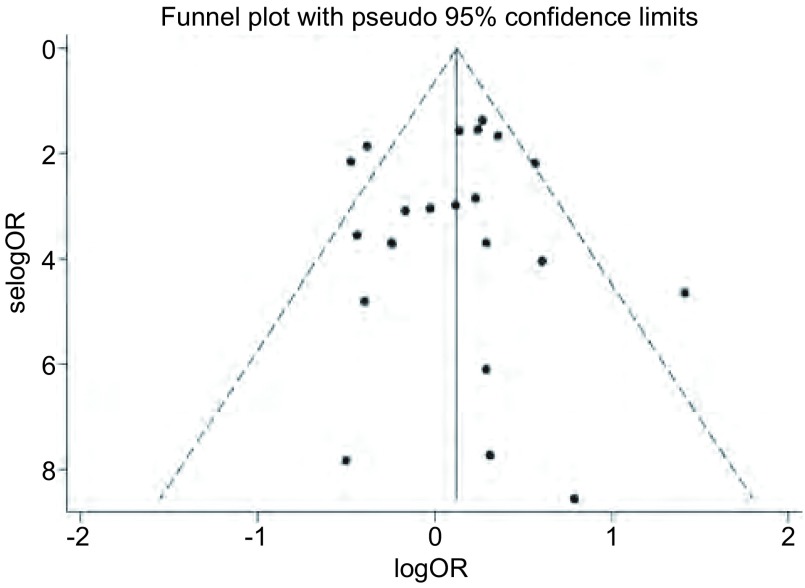
关于*hOGG1* Ser326Cys多态性与肺癌发病风险研究的漏斗分析图 Funnel plot analysis on the association of *hOGG1* Ser326Cys polymorphism with risk of lung cancer

## 3 讨论

肺癌是世界上最为常见的一种癌症，尽管人们认为吸烟是导致肺癌的一个主要风险因素，但是在终生吸烟的人群中，最终患肺癌的人不超过20%^[11]^，这说明遗传易感性对于癌症的发生起到重要的作用。

关于*hOGG1*基因，近年来分子流行病和遗传学研究主要集中在其第7外显子的Ser326Cys多态与癌症易感性的关系。然而关于Ser326Cys多态性与肺癌易感性是否相关尚存在争议，为了更好地探讨hOGG1多态性与肺癌易感性之间的关系，我们进行了*meta*分析。对纳入研究的22篇文献^[3, 4, 9, 10, 12-29]^进行综合分析，发现这些文献之间存在很强的异质性。对这些文献的对照组基因型数据进行*HWE*检验，有发现2篇文献^[9, 10]^的数据不符合*HWE*，将这两篇文献排除后，其余的20项研究^[3, 4, 12-29]^的结果则呈现出较好的同质性。这些结果提示我们，分析质量较高的研究结果有利于提高*meta*分析时数据的一致性。更为重要的是，我们发现在亚洲和混合型人群中，Ser326Cys多态与肺癌易感性存在明显正相关，Cys326基因型相比Ser326基因型能明显增加肺癌发病风险，这说明Ser326Cys多态与肺癌易感性之间的关系可能与种族差异有关。在亚洲或混合型人群中，Ser326Cys位点的改变更易导致肺癌发病风险的增加。同样，我们按照对照来源进行亚组分析，发现在人群来源和医院来源的研究中Cys326基因型相比Ser326基因型均明显增加了肺癌发病风险。

综上所述，为了更好地研究*hOGG1* Ser326Cys多态与肺癌易感性之间的关系，需要对大样本量进行人种、肺癌病理分型、地域环境、吸烟史等更细致的划分，这样才能准确地评估遗传因素与环境因素分别对肺癌发病风险的贡献。
